# Establishment and evaluation of retroperitoneal liposarcoma patient-derived xenograft models: an ideal model for preclinical study

**DOI:** 10.7150/ijms.70706

**Published:** 2022-07-11

**Authors:** Chang Xu, Liang Yan, Qiming An, Sha Zhang, Xiaoya Guan, Zhen Wang, Ang Lv, Daoning Liu, Faqiang Liu, Bin Dong, Min Zhao, Xiuyun Tian, Chunyi Hao

**Affiliations:** 1 Key Laboratory of Carcinogenesis and Translational Research (Ministry of Education/Beijing), Department of Hepato-Pancreato-Biliary Surgery, Peking University Cancer Hospital & Institute, Beijing, China; 2 Department of Gastrointestinal Surgery, the Affiliated Hospital of Inner Mongolia Medical University, Hohhot, China; 3 Department of Critical Care Medicine, Shandong Provincial Hospital Affiliated to Shandong First Medical University, Jinan, Shandong, China; 4 Key Laboratory of Carcinogenesis and Translational Research (Ministry of Education), Central Laboratory, Peking University Cancer Hospital & Institute, Beijing, China; 5 Key Laboratory of Carcinogenesis and Translational Research (Ministry of Education), Department of Pathology, Peking University Cancer Hospital & Institute, Beijing, China

**Keywords:** Retroperitoneal liposarcoma, patient-derived xenograft (PDX), prognosis, treatment evaluation, tumor biology research

## Abstract

Retroperitoneal liposarcoma (RLPS) is one of the most common subtypes of retroperitoneal soft tissue sarcomas. It is characterized by poor sensitivity to radiotherapy and chemotherapy and a low success rate of complete surgical resection. However, there are few reliable preclinical RLPS models for target discovery and therapy research. In this study, we aimed to establish RLPS patient-derived xenograft (PDX) models that are useful for biological research and preclinical drug trials. A total of 56 freshly resected RLPS tissues were subcutaneously transplanted into non-obese diabetic-severe combined immune deficient (NOD-SCID) mice, with subsequent xenotransplantation into second-generation mice. The tumor engraftment rate of first generation PDXs was 44.64%, and higher success rates were obtained from implantations of dedifferentiated, myxous, pleomorphic, high-grade liposarcomas and those with retroperitoneal organ infiltration. The first- and second- generation PDX models preserved the histopathological morphology, gene mutation profiles and MDM2 amplification of the primary tissues. PDX models can also provide the benefit of retaining original tumor biology and microenvironment characteristics, such as abnormal adipose differentiation, elevated Ki67 levels, high microvessel density, cancer-associated fibroblast presence, and tumor-associated macrophage infiltration. Overall survival (OS) and disease-free survival (DFS) of patients with successful first-generation PDX engraftment were significantly poorer than those with failed engraftment. Treatment with MDM2 inhibitor RG7112 significantly suppressed tumor growth of DDLPS PDX in mice. In conclusion, we successfully established RLPS PDX models that were histologically, genetically, and molecularly consistent with the original tissues. These models might provide opportunities for advancing RLPS tumor biology research, facilitating the development of novel drugs, particularly those targeting MDM2 amplification, adipose differentiation process, angiogenesis, cancer-associated fibroblasts, and so on.

## Introduction

Retroperitoneal soft tissue sarcomas (RSTSs) are a group of rare malignant tumors that arise in the retroperitoneum and mainly derive from fat, loose connective tissue, muscle, blood vessels, and other tissues in the retroperitoneum 1. Retroperitoneal liposarcoma (RLPS) is the most common RSTS and accounts for approximately 45% of all RSTSs 2,3. RLPS is further classified by morphological and genetic characteristics into well-differentiated liposarcoma (WDLPS), dedifferentiated liposarcoma (DDLPS), myxoid liposarcoma (MLPS), and pleomorphic liposarcoma (PLPS) 4.

At present, the most effective treatment of RLPS is surgical resection. However, for RLPS patients, complete resection and negative surgical margins are challenging due to occult onset, complex anatomical location, and large tumor volume. Outcomes of patients with positive surgical margins are similar to those patients who do not undergo surgical resection 5. Furthermore, results from radiotherapy and traditional chemotherapy are discouraging for RLPS patients. A previous study showed that perioperative radiotherapy of local RLPS might increase local recurrence rate 6. Adriamycin and Adriamycin-ifosfamide combination chemotherapies are standard treatment in advanced liposarcomas (LPS) 7. But only a small proportion of RLPS patients achieve objective response to these treatment regimens, and adverse reactions increase throughout prolonged progression-free survival (PFS) 8. Recent preclinical studies and clinical trials of novel LPS treatments are promising. The chemotherapeutic drugs trabectedin and eribulin are shown to be effective in treating advanced LPS and have been approved by FDA (Food and Drug Administration) as second-line therapies 9,10. MDM2 and CDK4 inhibitors have shown efficacy in maintaining long-term disease stability in WDLPS/DDLPS patients 11-13. Immune checkpoint inhibitors are another effective treatment option. In a phase II pembrolizumab clinical trial involving 10 DDLPS patients, two patients achieved partial remission, while four reached stable disease 14. Targeted therapies and immunotherapy have been proved as viable options for LPS treatment. But due to the relatively low incidence, clinical trials are limited by small sample sizes (typically between 20-60 cases), and they often don't cover all LPS subtypes. In addition, research on novel tumor treatment often doesn't include LPS cases. To overcome these obstacles, it is critical to construct stable and effective preclinical RLPS models including all pathological subtypes.

Tumor cell lines cultured* in vitro*, cell line-derived xenografts (CDXs) and patient-derived xenografts (PDXs) are currently used to predict the effect of anti-cancer agents. As for cell lines and CDXs, structure and heterogeneity of original tumors are somewhat lost, especially the tumor microenvironment. So the therapeutic effect on them cannot effectively represent the clinical outcome. PDXs are established by transplanting human tumor tissues into immunodeficient mice, and can preserve nearly all histological and molecular features of the original tumors 15. Due to the accurate reproduction of essential tumor microenvironment components, PDXs can maintain tumor heterogeneity and act as reliable tools for drug response prediction. The aim of our study was to transplant RLPS PDXs and to characterize the histologic, genetic, and molecular features of multi-generation PDX models and corresponding primary tumors. We also analyzed the potential influencing factors of PDX construction, the correlation of PDX construction with RLPS prognosis and the therapeutic response of DDLPS PDX models to MDM2 inhibitor RG7112. This study can provide a reliable *in vivo* preclinical model for the rare RLPS, so as to facilitate new treatment assessment and biology research of RLPS.

## Materials and methods

### Patients and tumor samples

Tumor samples were collected from 56 RLPS patients who underwent surgery at Peking University Cancer Hospital between 2015 and 2021. These patients were pathologically diagnosed as RLPS and did not receive chemotherapy or radiotherapy prior to surgery. Clinicopathological data were acquired from medical records, and written informed consent was obtained from each patient. This study was approved by the Institutional Review Board of Peking University Cancer Hospital (2019KT19).

Passage 0 (P0) tumor samples harvested from RLPS patients were cut into pieces with diameter of 5-10 mm. Tissues without obvious hemorrhage and necrosis were placed in fetal bovine serum (FBS)-free 1640 medium (Gibco, USA) and transferred to the Experimental Animal Center of Peking University Cancer Hospital for PDX construction. Other tissues were divided into three parts: one part was cryopreserved in 90% FBS/10% DMSO for later resuscitation; one part was fixed in 4% formalin for future histological analysis, and the third part was snap-frozen in liquid nitrogen and transferred to -80°C for RNA or total protein protection.

### Establishment of PDX models

P0 tumor tissues with a diameter of 3-5 mm were implanted bilaterally into the lower back of 5- to 6-week-old female NOD-SCID mice. Tumor size of initial recipients (Passage 1, P1) was measured with a vernier caliper once a week, and tumor volume was calculated using the formula: 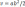 (a, tumor length; b, tumor width). When the volume of each P1 tumor reached approximately 1000 mm^3^, the mouse was euthanized, and the tumor was harvested and preserved in the same manner as P0 tissue and subsequently transplanted into Passage 2-4 (P2-4) mice. All mice were housed in specific-pathogen-free conditions with controlled ambient temperatures of 22-26°C and relative humidity of 40%-60% and provided unencumbered access to food and water. All studies were conducted in compliance with the animal ethics and welfare requirements as specified in the corresponding sections of *Guidelines for the Care and Use of Laboratory Animals of the National Institutes of Health* (ethics approval number: EAEC2018-06).

### Histological characteristics, genetic features and molecular expression of original tumors and PDX tissues

Formalin-fixed tissues were embedded in paraffin and cut into 4 μm thickness for hematoxylin and eosin (HE) and immunohistochemistry (IHC) staining. The following antibodies were used for IHC staining in primary tumors and corresponding PDX tumors: anti-PPARγ (#2435, 1:800, Cell Signaling Technology, Danvers, MA, USA), anti-CD163 (ZM-0428, working solution, ZSGB Biotech, Beijing, China), anti-Ki67 (ab15580, 1:100, Abcam, Cambridge, MA, USA), anti-CD34 (ab81289, 1:200, Abcam, Cambridge, MA, USA), anti-α-SMA (ab7817, 0.034 μg/ml, Abcam, Cambridge, MA, USA). The HE and IHC staining results were assessed by two independent pathologists.

Genomic DNA was extracted from both the tumor and the whole blood of a single patient (Case702, DDLPS) selected from the 56 donor patients. The former extraction was conducted using EasyPure Genomic DNA Kit (Transgen Biotech, Beijing, China) and the latter, which was used as a control, with a whole blood genomic DNA rapid extraction kit (BioTeke, Wuxi, China), both according to the manufacturers' instructions. High throughput whole-exome sequencing (WES) of 277 tumor-related genes was performed using the quality-tested DNA. The original sequence was evaluated against the human reference genome, and the sequencing depth and coverage depth of the target area were analyzed. Single-nucleotide polymorphisms and insertion/deletion mutations were analyzed, and multiple databases were used to annotate the mutation results.

To assess *MDM2* specific gene amplification, fluorescence *in situ* hybridization (FISH) was performed on paraffin-embedded sections of tumor tissue and corresponding PDX models (P1-P4) of patient Case702, using the Vysis MDM2/CEP12 FISH Probe Kit (Abbott, Abbott Park, IL, USA), according to the manufacturer's instructions.

Total RNA was extracted from normal fat, tumor tissue and corresponding PDX models (P1-P2) using TRIzol reagent (Thermo Fisher Scientific, Inc.). To evaluate the mRNA expression of *MKI67*, *PPARγ*, *CEBPα*, *LPL*, and *ADIPOQ*, RT-qPCR was performed, with the following primers: *MKI67*, 5′-ACGCCTGGTTACTATCAAAAGG-3′ (forward) and 5′-CAGACCCATTTAC-TTGTGTTGGA-3′ (reverse); *PPARγ*, 5′-TACTGTCGGTTTCAGAAATGCC-3′ (forward) and 5′-GTCAGCGGACTCTGGATTCAG-3′ (reverse); *CEBPα*, 5′-CAAGAACAGCAACGAGTACCG-3′ (forward) and 5′-GTCACTGGTCAACTC-CAGCAC-3′ (reverse); *LPL*, 5′-AGGATGTGGCCCGGTTTATC-3′ (forward) and 5′-CCAAGGCTGTATCCCAAGAGAT-3′ (reverse); *ADIPOQ*, 5′-AACATGCCCATT-CGCTTTACC-3′ (forward) and 5′-TAGGCAAAGTAGTACAGCCCA-3′ (reverse).

### Evaluation of the therapeutic response of PDX model to preclinical drug

A group of mice bearing DDLPS PDXs with MDM2 amplification (case702) were used for drug sensitivity assay of MDM2 inhibitor RG7112 (APE×BIO, USA). When the P4 PDX tumors were nearly 40mm^3^, 10 tumor-bearing mice were randomly divided into two groups. Mice in the experimental group were treated with RG7112 daily by oral gavage at a dosage of 25mg/kg, for 14 days. The control group was given the same volume of 1% CMC Na. Body weight and tumor volume of the mice were measured at least twice a week. After drug withdrawal, mice were observed for another 20 days and then sacrificed.

### Statistical analysis

GraphPad Prism 8 and SPSS 21.0 were used for statistical analysis. Pearson's χ^2^ test was used to analyze the correlation between clinicopathological characteristics and PDX engraftment rate. Mann-Whitney test was used to compare mRNA expression of normal fat and that of primary tumor or PDX model tissues. Kaplan-Meier survival analysis was performed on overall survival (OS) and disease-free survival (DFS) assessments in 38 RLPS patients, and the difference in OS and DFS was calculated using log-rank test. Statistical significance was set at P<0.05.

## Results

### Characteristics of RLPS patients that were used to construct PDXs

Tumor samples from 56 RLPS patients were obtained to establish PDX models. The median age was 55 years (range:30-74), wherein 34 (60.7%) patients were male and 22 (39.3%) were female. Among the 56 cases, 35 (62.5%) were DDLPS, 16 (28.5%) were WDLPS, 3 (5.4%) were PLS, and 2 (3.6%) were MLPS. Thirty-eight (76.0%) cases were of medium or high grade, and 12 (24.0%) cases were of low grade. In addition, 36 (64.3%) cases were of retroperitoneal organ infiltration. The primary cases and recurrent cases were 28 respectively (50%).

### PDXs establishment and influencing factors analysis

As shown in Table 1, P1 PDX models were successfully established from 25 of 56 donor tissues (44.64%). And 14 P1 PDXs were transplanted into second generation (P2) mice, wherein 10 P2 PDXs were successfully implanted, representing a 71.43% transplantation rate. Engraftment rates of P2 were improved compared to P1, but the difference was not statistically significant.

Next, we analyzed the possible correlation of clinicopathological characteristics with engraftment rate of primary tumor tissue. As shown in Table 2, we can see that transplantation rate of P1 PDXs was related to RLPS pathological subtype (P=0.013), tumor grade (P=0.001), and tumor organ invasion (P=0.048). Engraftment rates were higher in DDLPS, MLPS and PLS patients, compared with WDLPS. High-grade RLPS, and RLPS with retroperitoneal organ infiltration were more readily to form P1 PDXs. In this group of PDX, the engraftment rate did not correlate with patient sex and age, or with tumor size, vascular invasion, lymph node metastasis and tumor site, or primary/recurrent status.

### Comparison of histopathological characteristics between primary tumors and PDXs

**Figure 1:**
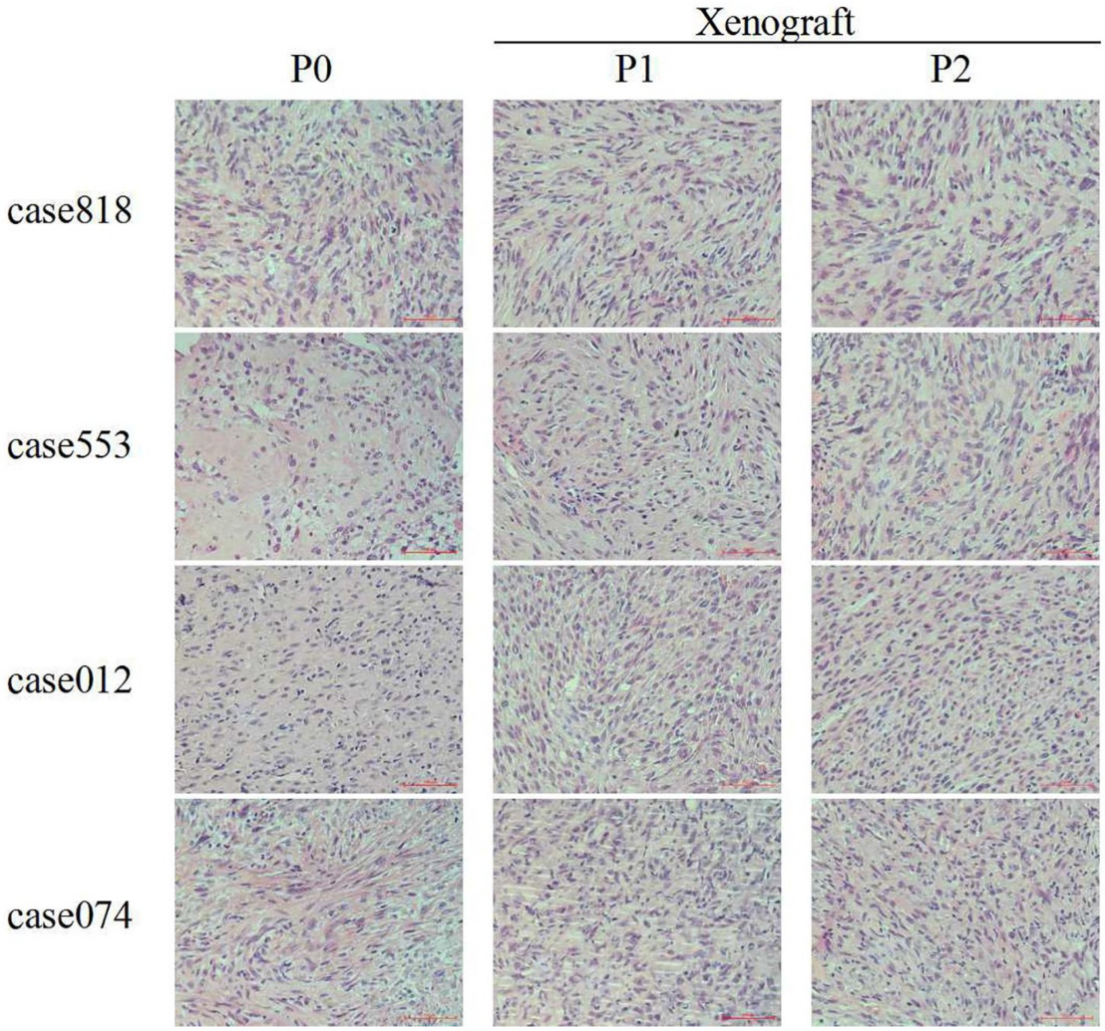
HE staining of RLPS tissues and corresponding P1-P2 PDX tissues. For DDLPS case 818, case 553, case 012 and case 074, original tumor (P0) and corresponding P1-P2 PDX tissues showed similar morphological structure. Scale bars, 100 μm.

The histology and grading of primary tumors and corresponding P1/P2 PDXs were reviewed by two independent pathologists. HE staining of the original tumors and corresponding PDX tissues showed similar morphological structure and consistently high grade. These results demonstrated that histological characteristics and pathological pattern were maintained during serial passaging (Figure 1, Table 3).

### Comparison of genetic features between primary tumors and PDXs

**Figure 2:**
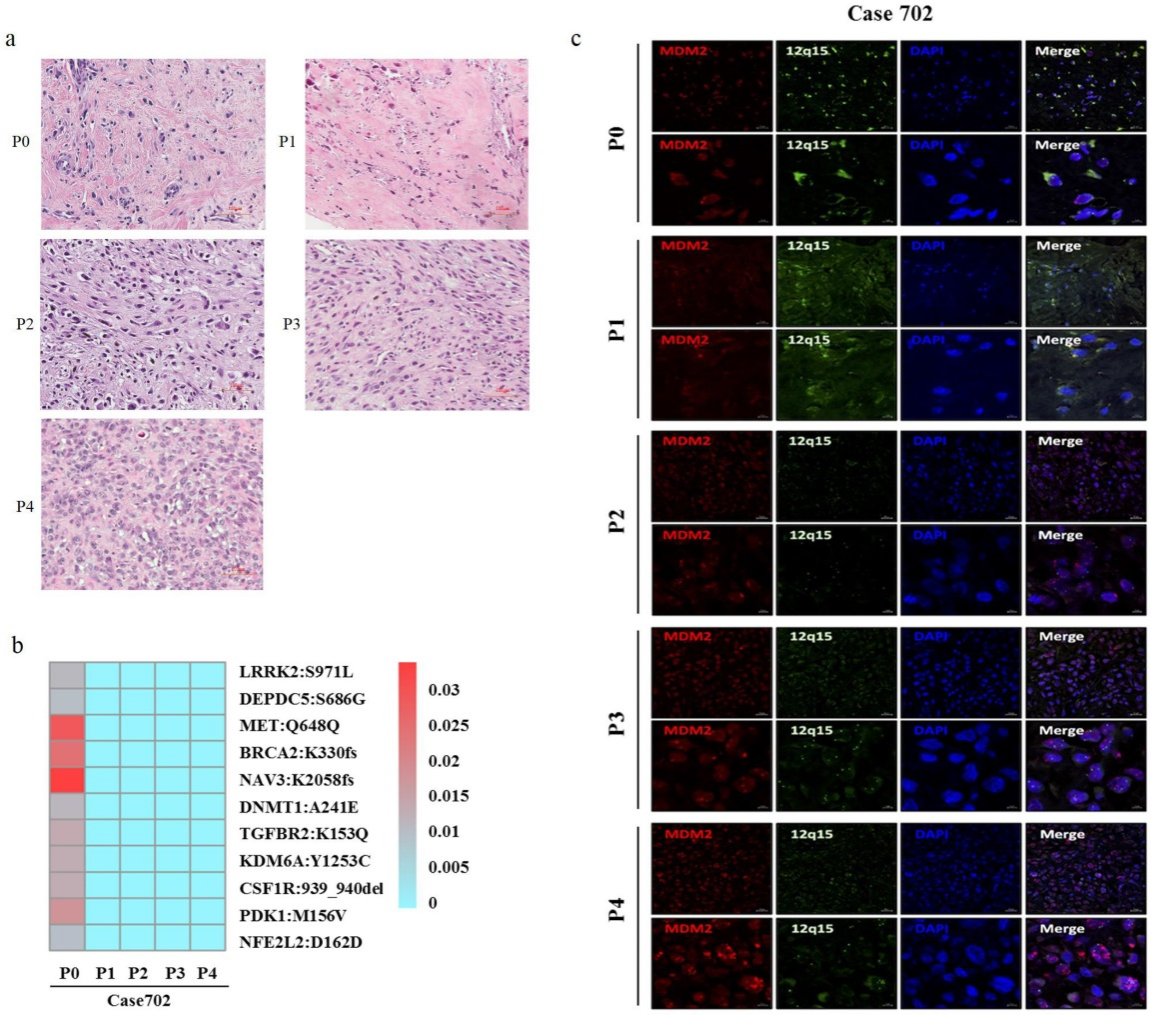
Genetic features of primary tumors and corresponding P1-P4 PDXs in Case702. (a) HE staining of Case702 and corresponding PDXs. Scale bars, 100 μm. (b) In Case702, low-frequency gene mutations with the highest frequency not exceeding 0.03 was detected in the original tumor (P0), and no additional obvious mutations of these genes could be detected in P1-P4 PDXs. (c) FISH analysis showed that both P0 tumor and P1-P4 PDXs had MDM2 amplification (red fluorescence). Green fluorescence indicated chromosome 12, blue fluorescence showed the nucleus stained by DAPI. Scale bars, 20 μm and 5μm.

In order to assess the effects of serial passaging on genetic stability, high throughput WES was used to analyze 277 high-frequency gene mutations in the primary tumor and corresponding P1-P4 PDX tumors of patient Case702, whose whole blood DNA was used as a control. HE staining has shown that Case702 primary tumor and corresponding PDXs have shown consistently histological characteristics (Figure 2a). Eleven mutation sites were detected in patient tumor tissues, including *NAV3*, *MET*, *BRCA2*, and *PDK1*. The mutation frequency was relatively low, and the highest frequency did not exceed 0.03. Mutations in these genes were not detected in corresponding P1-P4 PDXs (Figure 2b, Table 4). These results indicated that the PDX models replicated the original gene mutation spectrum, and no additional obvious mutation sites were detected in PDX tissues.

FISH visualization was used to detect MDM2 amplification in the primary tumor and P1-P4 PDXs of patient Case702. As illustrated in Figure 2c, red fluorescence indicated MDM2 gene, green fluorescence indicated chromosome 12, and blue fluorescence showed the nucleus stained by DAPI. Multiple red signal clusters could be seen in the tumor cells of all tissues, suggesting MDM2 amplification in primary tumor and PDXs tissues. This phenomenon was consistent with the characteristic of DDLPS, which confirmed the genetic fidelity of original tumor in PDX models.

### Comparison of molecular expression between primary tumors and PDXs

**Figure 3:**
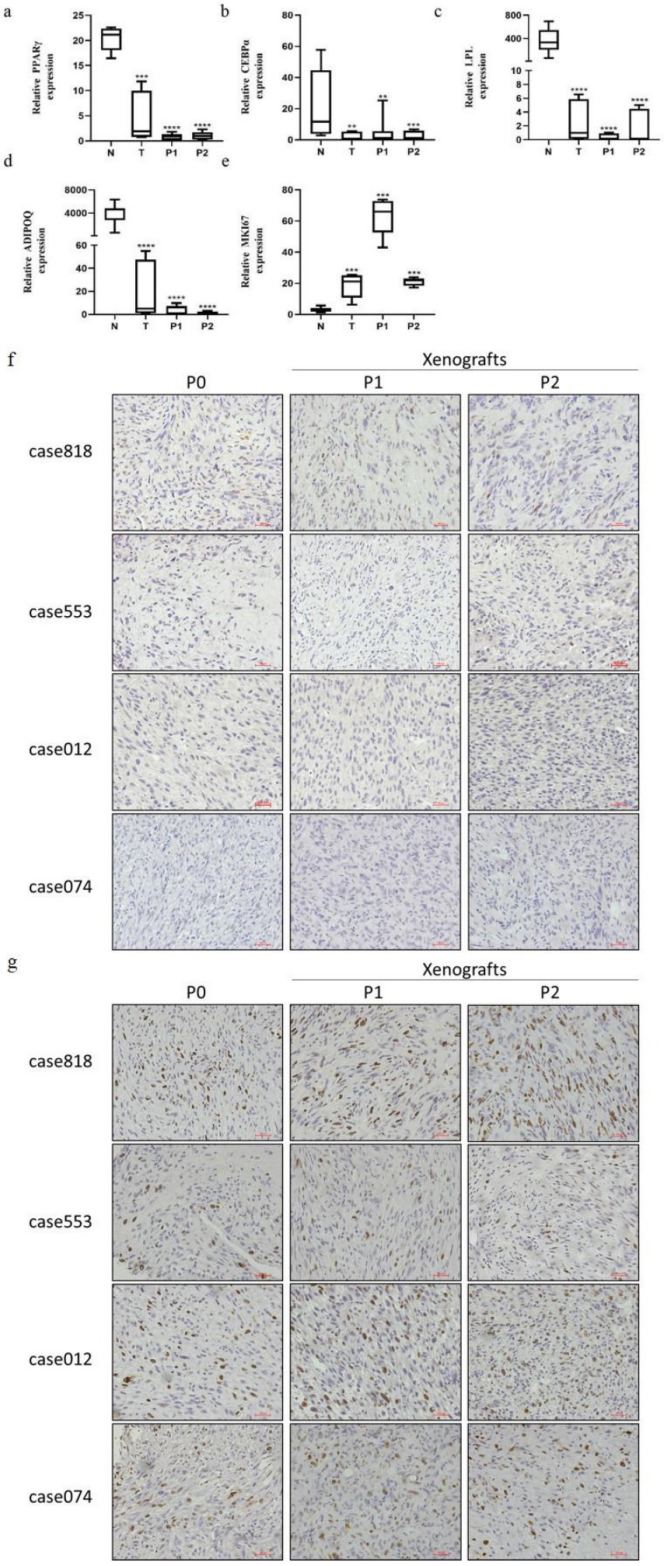
Expression of adipogenic differentiation and proliferation markers in fat, original RLPS tissues and corresponding P1-P2 PDX models. (a-e) Relative mRNA expression levels of *PPARγ*, *CEBPα*, *LPL*, *ADIPOQ*, and *MKI67*. (f) Representative IHC staining of PPARγ. P0 and P1-P2 PDX models are of PPARγ low expression. (g) Representative IHC staining of Ki67. P0 and P1-P2 PDX models are of Ki67 high expression. **P<0.01, ***P<0.001, ****P<0.0001 compared with normal fat. Scale bars, 100 μm.

Due to abnormal adipogenesis, adipogenic differentiation markers and mature adipose specific secretions are absent in RLPS. Based on this, we compared the mRNA expression of *PPARγ*, *CEBPα*, *LPL* and *ADIPOQ* in ten normal adipose tissues and four primary tumors and their corresponding P1 and P2 PDX tumors (Figure 3a-d). Messenger RNA expression of *PPARγ*, *CEBPα*, *LPL*, and *ADIPOQ* in the primary tumors and corresponding PDX tumors were consistently lower than those of normal fat (P<0.05). In addition, consistent with the primary tumor, P1 and P2 PDX models also maintained elevated *MKI67* mRNA expression (Figure 3e).

**Figure 4:**
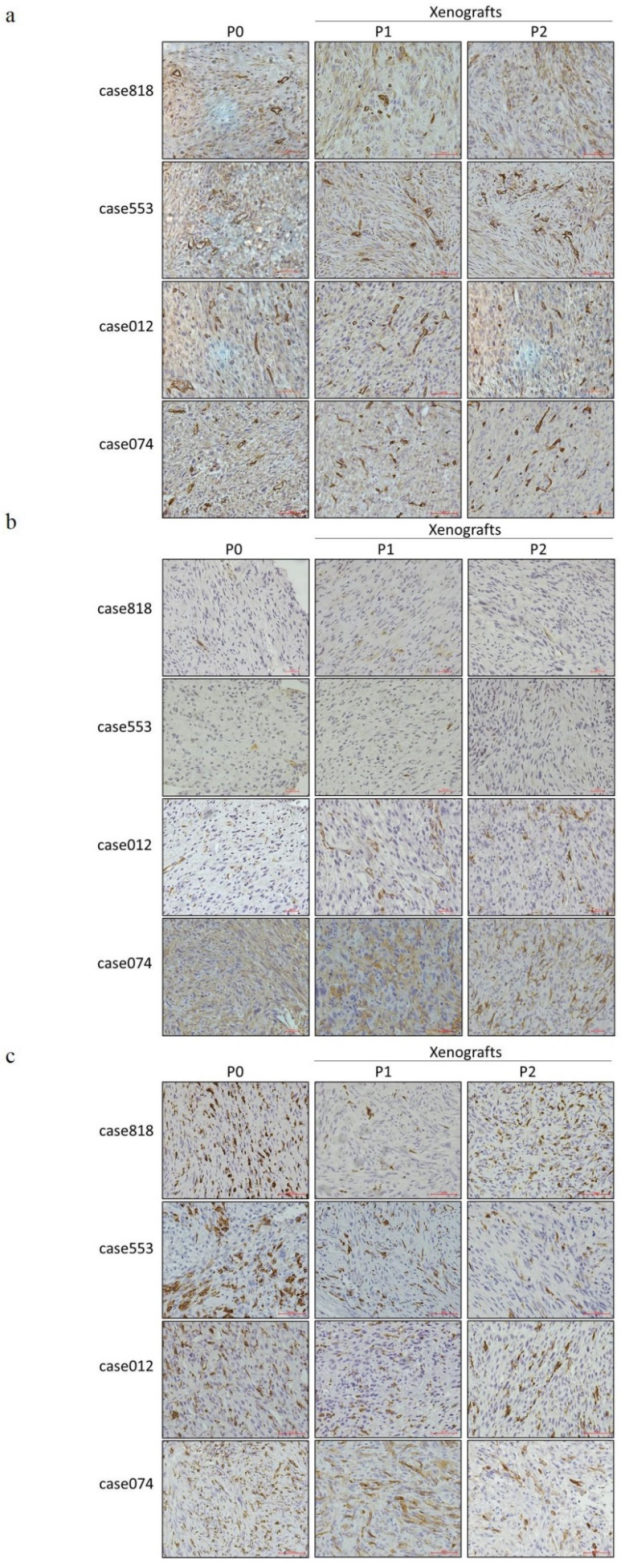
Expression of tumor microenvironment components in RLPS tissues and corresponding P1-P2 PDX models. (a) Representative IHC staining of CD34. P0 and P1-P2 PDX models are of high microvessel density. (b) Representative IHC staining of α-SMA. P0 and P1-P2 PDXs are both of CAFs infiltration. (c) Representative IHC staining of CD163. P0 and P1-P2 PDXs are both of TAMs infiltration. Scale bars, 100 μm.

IHC staining was used to evaluate PPARγ, an important transcription factor in the regulation of adipogenic differentiation, and Ki67, an antigen associated with cellular proliferation, in primary tumors and corresponding PDX tumors (Figure 3f-g). Staining results indicated that the patient tumors and corresponding xenografts showed similar expression patterns of characteristic markers: low PPARγ level and high Ki67 level. We also performed CD34 staining to assess microvessel density, α-SMA staining to label cancer-associated fibroblasts (CAFs), and CD163 staining to label tumor-associated macrophages (TAMs). Both P1 and P2 PDX models reproduced the tumor microenvironment of the primary tumor, replicating the high microvessel density and CAFs/TAMs infiltration (Figure 4a-c).

### Correlation between engraftment of P1 PDX models and survival of RLPS patients

**Figure 5:**
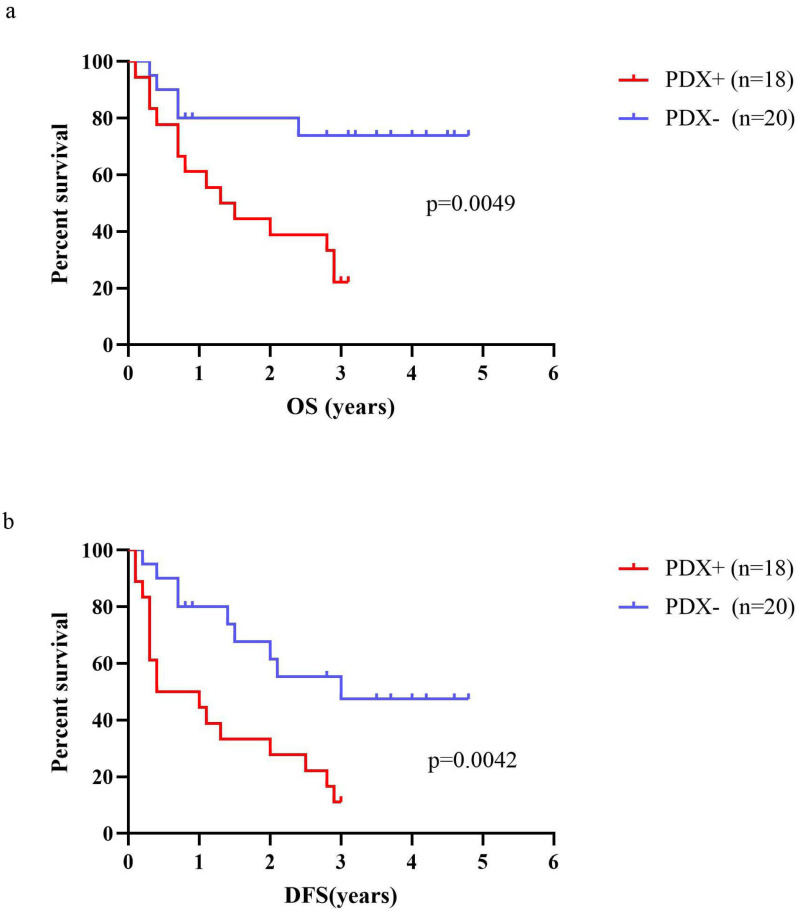
Correlation of OS or DFS of RLPS patients with engraftment status of RLPS tissues. (a) OS in RLPS patients with successful P1 PDXs transplantation was poorer than that with failed P1 PDXs transplantation (1.7 years vs. 2.6 years, p=0.0049). (b) DFS in RLPS patients with successful P1 PDXs transplantation was poorer than that with failed P1 PDXs transplantation (1.2 years vs. 2.2 years, p=0.0042).

A total of 38 RLPS patients were evaluated for OS and DFS. The median OS of RLPS patients with successful engraftment in mice was 1.7 years (range: 0.1-3.1 years), and the median DFS was 1.2 years (range: 0.1-3.0 years). For the patients with failed transplantation, the median OS was 2.6 years (range: 0.3-4.8 years) and the median DFS was 2.2 years (range: 0.2-4.8 years). Log-Rank test showed that the OS and DFS in patients with successfully transplanted tumors was poorer than those with failed P1 PDXs transplantation (P=0.0049, Fig 5a; P=0.0042, Fig 5b). Therefore, the transplantation status of P1 PDX might indicate the postoperative survival of RLPS patients.

### RLPS PDXs were an ideal model for preclinical study

**Figure 6:**
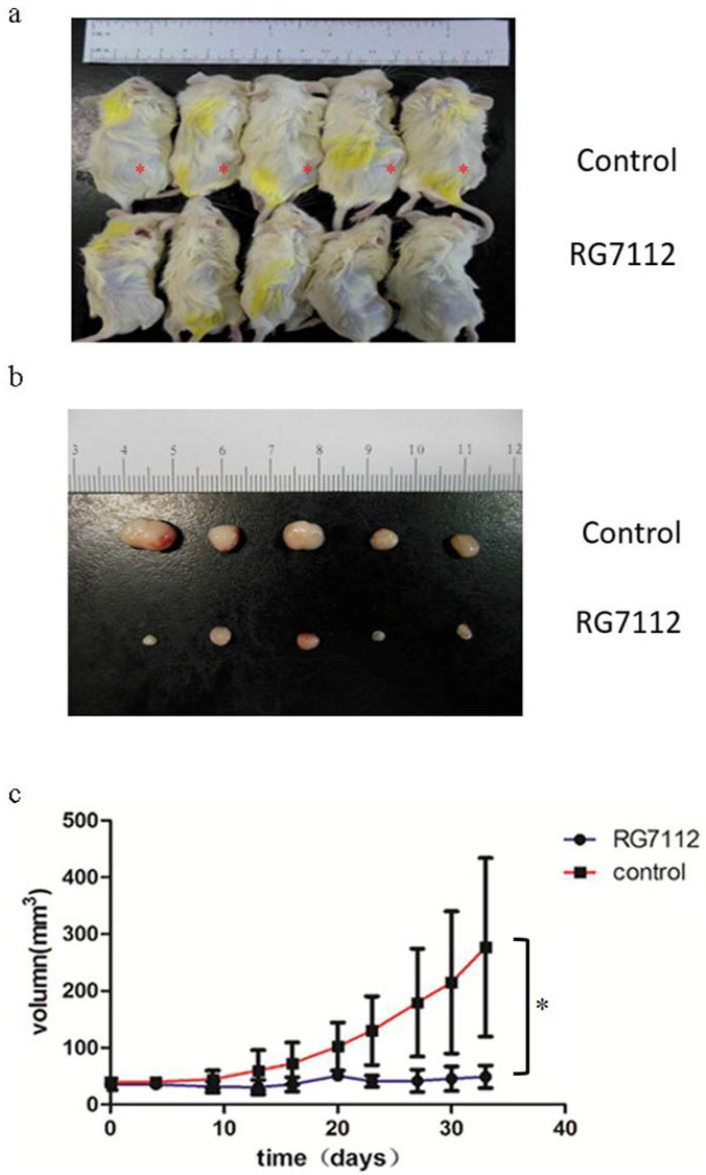
Therapeutic response of PDX (Case702) to MDM2 inhibitor RG7112. (a-b) Compared with control group, the tumor volume of mice in treatment group were significantly reduced.*:PDX tumors. (c) The mice treated with RG7112 had a lower PDX growth rate (p=0.015).

Next, we explored the potential use of RLPS PDX as a preclinical model. We selected Case702 with significant MDM2 amplification to explore whether MDM2 inhibitor RG7112 can inhibit the growth of DDLPS PDXs. Ten P4 PDX-bearing mice were randomly divided into two groups, which were treated with RG7112 or 1% CMC Na by oral gavage respectively. The tumor volume and mouse weight were measured twice a week. Following drug withdrawal, the mice were observed for a period of 20 days, and then they were sacrificed and photographed (Figure 6a). From Figure 6(b) and 6(c), we can see that compared with the control group, the tumor volumes of mice in RG7112 group were significantly reduced, and the mice treated with RG7112 had a lower PDX growth rate (p=0.015). Therefore, the PDX models we constructed could predict the patients' treatment response to preclinical drugs.

## Discussion

According to the World Health Organization, soft tissue sarcomas (STSs) are classified into 11 types and 176 subtypes. STSs of different histological types display strong heterogeneity with diverse genetic and molecular profiles, and respond differently to conventional chemotherapy, targeted therapy and immunotherapy 16. However, due to the relatively low incidence of STSs, approximately 1% of adult malignant tumors 6, few clinical studies focused on specific pathological subtypes of STS, so the results on a certain subtype are often negative or non-significant. In the present study, we investigated LPS, the most common type of STS.

LPS located at different anatomical sites have different clinical behaviors, biological characteristics, and treatment responses. For example, when WDLPS occurs in the retroperitoneal space, mediastinum, or the area around the testis, local recurrence is more likely and often leads to patient death 17. LPS located in the extremities are often successfully resected with negative surgical margins. However, the optimal range of RLPS resection is still controversial, and extensive resection to reduce local recurrence is viewed as an option 18,19. Furthermore, LPS of the limbs is treated with adjuvant therapy or neoadjuvant radiotherapy, but in contrast, perioperative radiotherapy might increase the local recurrence rate of RSTS 6. In light of these limitations, we chose to focus our research on RLPS, which is difficult to be completely resected, prone to recurrence, and resistant to chemotherapy or radiotherapy. At present, stable and effective preclinical models of RLPS have not been reported. Animal models that can accurately simulate human tumors and reproduce the tumor microenvironment and heterogeneity are essential to promote the exploration of new treatments for RLPS. PDX model is to directly transplant patient-derived tumor tissue into the subcutaneous of immune deficient mice, so that the structure, molecular expression and biological characteristics of the original tumors can be maintained. Now, PDXs have been used to evaluate the effects of anti-cancer drugs and to investigate drug resistance mechanisms 15.

In this study, we described and characterized a group of RLPS PDXs for the first time. Freshly resected tumors from 56 RLPS patients were subcutaneously transplanted into NOD/SCID mice, successfully producing 25 first generation PDX models. And then, 10 P2 PDXs were successfully implanted out of 14 P1 PDXs. The transplantation success rates of P1 and P2 PDXs were 44.64% and 71.43%, respectively. A previous study of 188 STS PDXs showed that the engraftment rate was 32%, lower than our results 20. This difference may be due to our selection of NOD/SCID mice with more severe immunosuppression, and fresh tumor tissues from surgical resection rather than biopsy, which avoids transplantation failure caused by insufficient tissue volume. Another STS PDX study reported a higher engraftment rate of 76% 21, wherein only 5 of the 29 STSs were LPS. In addition, they distributed each primary tumor tissue into multiple mice. However, we only implanted each donor tissue into one NOD/SCID mouse bilaterally.

In addition to NOD/SCID mice and surgically resected tumor tissues are helpful to the construction of PDX, we also carefully analyzed the factors that might affect the formation of P1 PDXs. PDX engraftment rates were higher in patients with DDLPS, MLPS, PLPS, high-grade RLPS, and RLPS with parenchymal infiltration of retroperitoneal organs such as the pancreas, kidney, or spleen. This suggests that such patients are more likely to benefit from the PDX models. Interestingly, parenchymal organ infiltration was a better indicator of successful xenografts than tumor size, presumably because it reflected a high degree of malignancy and strong invasiveness. This finding is consistent with our previous study that organ parenchymal infiltration was an independent risk factor for DFS in patients with primary RLPS (P=0.039) (unpublished data). The aforementioned study of 188 STS PDXs did not find factors affecting successful PDX engraftment 20, which might be attributed to mixed clinicopathological characteristics of patients with different sarcoma subtypes.

Our team also built a group of PDX models for pancreatic ductal adenocarcinoma (PDAC) and hepatocellular carcinoma (HCC) using similar methods 22,23. In contrast to our findings in RLPS, no significant correlation was found between PDAC PDX engraftment rate and clinicopathological features or prognosis. But consistent with the HCC PDX study, successful RLPS PDX engraftment was also correlated with poorer prognosis (the shorter OS and DFS). Similarly, in breast cancer and non-small cell lung cancer, patients with successfully transplanted PDXs had worse OS 24,25. Therefore, the successful establishment of RLPS PDXs is correlated with specific clinicopathological characteristics and postoperative survival of patients, thus it is a good research model with regularity and clinical significance.

What's more, HE staining revealed that the PDX models replicated the histological structure, pathological characteristics, and high tumor grade of the primary tumors. High-throughput WES and FISH indicated that PDX tissues also possessed the same gene mutation profile and MDM2 amplification as the original tumors. Therefore, the histopathological characteristics and genetic features are highly consistent with the original tumors throughout PDX passaging. It is known that MDM2 is the signature amplified gene and potential therapeutic target of WD/DDLPS. So RLPS PDXs could be used to assess safety and efficacy of MDM2 antagonists. Other targets such as the amplified genes CDK4 and HMG2A in WD/DDLPS, the fusion gene FUS-DDIT3 in MLPS can also be further identified in RLPS PDXs. These genetically stable PDXs can provide an effective platform for targeted therapy research.

Compared with normal fat, PDX tissue displayed lower expression of adipose-related molecules and elevated presence of Ki67, replicating the molecular expression of primary tissues. Recently insight into adipogenesis revealed that therapies promoting adipogenesis in LPS has theoretical advantages over other tumors. PPARγ and C/EBPα play a central role in adipogenesis by regulating the expression of genes involved in adipocyte maturation 26. PPARγ and C/EBPα are downregulated in WD/DDLPS 27, and the *FUS-CHOP* fusion gene in MLPS can inhibit their expression, leading to the abnormal proliferation and accumulation of undifferentiated adipocytes 28. Our study found that, consistent with the molecular profile of the corresponding primary tumors, levels of PPARγ, CEBPα, and mature adipocyte-specific products LPL and ADIPOQ were significantly lower in RLPS PDX model tissue than in normal fat. These outcomes suggest that RLPS PDX models can be used for PPARγ agonists and other adipogenic treatments. For instance, Frapolli proved that the PPARγ agonist pioglitazone can overcome trabectedin resistance in MLPS 29, and the PDX models can further be used to investigate this effect on other RLPS subtypes wherein trabectedin has been found to be beneficial 30. Because there is no known RLPS specific marker, we selected Ki67 as a proliferation marker 31 to determine whether RLPS PDX models could maintain the proliferation capacity and malignancy of original tumors. The PDX model also effectively reproduced the tumor microenvironments of corresponding originating tissues, maintaining high microvessel density and CAFs and TAMs infiltration throughout serial passaging. CAFs and TAMs can secrete vascular endothelial growth factors, HIF, IL6, and other cytokines to promote angiogenesis 32. Abnormal growth of new blood vessels is a prerequisite for tumor growth and metastasis 33. CAFs and TAMs also promote tumor metastasis by secreting matrix metalloproteinase and facilitating tumor cell chemotaxis 34-36. The PDX models would benefit preclinical studies of treatments that target tumor vessels, CAFs, and TAMs, and identify the beneficial population by evaluating levels of different microenvironment composition. Relevant to this study, we are currently conducting research on tyrosine kinase inhibitors, a kind of small-molecule anti-angiogenesis drugs, and we have shown that they can inhibit angiogenesis and tumor growth in the RLPS PDX model presented here 37.

Finally, we assessed whether RLPS PDXs can predict the therapeutic effect of preclinical drugs. RG7112 is the first MDM2 inhibitor that entered clinical trial 38, and it can inhibit the cell viability of colon cancer and osteosarcoma cell lines with wild-type p53 39. A phase I clinical trial showed that after RG7112 treatment, one patient achieved partial response and 14 patients achieved stable disease in the 20 WD/DDLPS patients who did not receive chemotherapy previously 40. Although MDM2 inhibitors have shown potential clinical application in LPS, the objective response rate is unsatisfactory. In our study, P4 xenograft tumors derived from patient Case702 had the same MDM2 amplification as the original tumor, and its growth can be inhibited by RG7112. The treatment response of RLPS PDXs to RG7112 is consistent with the results of clinical trial, suggesting that these PDX models can facilitate the application of preclinical drugs and predict the efficacy of different patients. Based on this, we can conduct further research such as the combination of MDM2 inhibitors with other drugs, screening beneficial patients, which will contribute to the personalized tumor treatment, especially for RLPS patients.

PDX models based on NOD/SCID mice also have certain limitations. NOD/SCID mice lack T lymphocytes, B lymphocytes, natural killer cells, and circulating complement components 41. Therefore, our PDX models cannot be used to assess the effect of treatments on the infiltration and function of these immune cells and cannot fully demonstrate the interaction mechanism between tumor cells and immune cells. An alternative solution is PDX-bearing mice using a humanized immune system. This animal model has a complete human-derived tumor immune system and can be transplanted with patient tumors for immunotherapy research 42. In future, we will explore RLPS PDX mouse models with human-derived immune systems to provide more valuable results for RLPS research.

## Conclusion

In summary, PDXs models of RLPS were successfully established and characterized for the first time. The histological, genetic, and molecular characteristics of PDXs are highly consistent with primary tumor tissues. Successful PDX engraftment is correlated with poorer OS and DFS of RLPS patients. PDX engraftment rate was higher in patients with DDLPS, MLPS, PLPS, high-grade RLPS, and RLPS with retroperitoneal organ infiltration, and such patients might benefit from research based on these models. PDX models established in this study can be used for biology research and new treatment evaluation of RLPS, especially for preclinical studies that target MDM2 amplification, adipogenesis process, tumor blood vessel or vessel formation, and CAFs and TAMs infiltration.

## Figures and Tables

**Table 1:** P1/P2 PDXs formation rate for all retroperitoneal liposarcoma types

	All RLPS (n=56)	WDLPS (n=16)	DDLPS (n=35)	MLPS (n=2)	PLS (n=3)
P1 PDXs					
No growth	31 (55.36%)	13 (81.25%)	18 (51.43%)	0	0
Success	25 (44.64%)	3 (18.75%)	17 (48.57%)	2 (100%)	3 (100%)
P2 PDXs					
No growth	4 (28.57%)	2 (100%)	2 (22.22%)	0	0
Success	10 (71.43%)	0	7 (77.78%)	2 (100%)	1 (100%)

**Table 2:** Correlations between engraftment rate and clinicopathological characteristics of all patients

Clinicopathological characteristics	No. of patients(%)	Successful engraftment (%)	P-value
**Gender**			0.412
Male	34 (60.7%)	17 (50.0%)	
Female	22 (39.3%)	8 (36.4%)	
**Age**			0.591
≥55	28 (50.0%)	14 (50.0%)	
<55	28 (50.0%)	11 (39.3%)	
**Tumor type**			0.013*
WDLPS	16 (28.5%)	3 (18.8%)	
DDLPS	35 (62.5%)	17 (48.6%)	
MLPS	2 (3.6%)	2 (100.0%)	
PLS	3 (5.4%)	3 (100.0%)	
**Tumor volume (cm^3^)**			>0.999
≥1000	35 (62.5%)	16 (45.7%)	
<1000	21 (37.5%)	9 (42.9%)	
**Grade**			0.001**
1	12 (24.0%)	3 (25.0%)	
2	18 (36.0%)	5 (27.8%)	
3	20 (40.0%)	16 (80.0%)	
**Vascular invasion**			0.462
Yes	1 (2.6%)	1 (100%)	
No	38 (97.4%)	17 (44.7%)	
**Lymph node metastasis**			>0.999
Yes	2 (4.5%)	1 (50.0%)	
No	42 (95.5%)	20 (47.6%)	
**Organ invasion**			0.048*
Yes	36 (64.3%)	20 (55.6%)	
No	20 (35.7%)	5 (25.0%)	
**Primary/recurrence**			0.591
Primary	28 (50%)	11 (39.3%)	
Recurrence	28 (50%)	14 (50%)	
**Distant metastasis**			>0.999
Yes	3 (7.7%)	1 (33.3%)	
No	36 (92.3%)	17 (47.2%)	

**Table 3:** Comparison of grade between 4 RLPS tissues and corresponding P1-P2 PDX tissues

Case	Patient original tumor (P0)	Xenograft	
		P1	P2
818	High	High	High
553	High	High	High
012	High	High	High
074	High	High	High

**Table 4:** Gene mutations in original tumor and xenografts of Case702

Gene	AA_change	Frequency				
		P0	P1	P2	P3	P4
*NAV3*	exon33: c.6172delA:p.K2058fs	0.03371	-	-	-	-
*MET*	exon7: c.1944A>G:p.Q648Q	0.02878	-	-	-	-
*BRCA2*	exon10: c.988delA:p.K330fs	0.02459	-	-	-	-
*PDK1*	exon4: c.466A>G:p.M156V	0.01802	-	-	-	-
*TGFBR2*	exon4: c.457A>C:p.K153Q	0.0137	-	-	-	-
*CSF1R*	exon22:c.2817_2819del:p.939_940del	0.01333	-	-	-	-
*KDM6A*	exon26: c.3758A>G:p.Y1253C	0.01316	-	-	-	-
*DNMT1*	exon9: c.722C>A:p.A241E	0.01176	-	-	-	-
*LRRK2*	exon23: c.2912C>T:p.S971L	0.01138	-	-	-	-
*DEPDC5*	exon23: c.2056A>G:p.S686G	0.01033	-	-	-	-
*NFE2L2*	exon4: c.486C>T:p.D162D	0.01005	-	-	-	-
